# Impact of Fish By-Product Hydrolysates, a Novel Food, on the Nutritional and Technological Properties of Fusilli Pasta

**DOI:** 10.1155/ijfo/8821646

**Published:** 2025-07-25

**Authors:** Paula Ardila, Adrián Honrado, Juan B. Calanche

**Affiliations:** Instituto Agroalimentario de Aragon-IA2-(Universidad de Zaragoza-CITA), Zaragoza, Spain

**Keywords:** bioactive peptides, by-product valorisation, circular economy, fish hydrolysate, functional pasta, Omega-3 fatty acids, sustainable food production

## Abstract

This study pioneers the innovative development and comprehensive characterisation of fusilli pasta enriched with protein hydrolysates derived from underutilised fish by-products (salmon, sea bass and tuna) materials typically discarded during seafood processing despite their significant nutritional potential. Fish hydrolysates were incorporated into durum wheat semolina at 1% and 3% concentrations to evaluate their impact on pasta quality and nutritional profile. Results demonstrated improvements in protein content and Omega-3 fatty acid profile across all enriched formulations compared to control samples. This incorporation significantly influenced technological properties, including optimal cooking time, weight gain, hydration capacity, cooking loss and colour parameters. Molecular weight fractionation revealed bioactive peptides with potential antioxidant properties, primarily in low molecular weight fractions. The microbiological assessment confirmed the absence of enterobacteria, moulds and yeasts in the final pasta products, although high viable microbial counts were detected in the raw hydrolysates. This research demonstrates the feasibility of valorising fish processing by-products to create innovative, nutrient-dense functional foods that align with contemporary consumer demands for sustainable production practices and enhanced nutritional profiles. Furthermore, it establishes a promising approach to circular economy principles within the by-product and pasta manufacturing sectors, potentially reducing waste whilst delivering products with improved health-promoting properties.

## 1. Introduction

Pasta represents one of the most significant staple foods worldwide, valued for its nutritional contribution, convenience and versatility. With a global production reaching approximately 14.4 million tonnes annually, it ranks amongst the most widely consumed foods in the world. This versatility is reflected in the variety of preparations and their availability in both dried and fresh forms [1]. The quality and cooking properties of pasta are closely related to the protein structure and starch network in the final product. The gluten network formation through protein interactions, particularly gliadin and glutenin proteins in durum wheat semolina, is essential for pasta texture and elasticity. When enriched with fish protein hydrolysates, the protein interactions in the dough may alter the gluten matrix, influencing factors such as texture, firmness and cooking behaviour [2]. These technological properties distinguish pasta as a unique and versatile food, evaluated through various parameters including optimal cooking time (OCT), weight gain, hydration, cooking loss and texture, which are determinants of its final quality [3]. For this reason, the composition of the raw material used in its production has a significant impact on its physical, chemical and textural properties [4].

In recent years, there has been a growing trend toward pasta products' nutritional enhancement, reflecting the broader movement in food science toward health-conscious alternatives and increasing functional foods and nutraceuticals [5]. Functional foods are recognised for their health-promoting properties and capacity to improve overall well-being beyond basic nutrition [6]. The term ‘functional food', which originated in Japan in the 1980s has been defined by the Food and Nutrition Board of the National Academy of Sciences as a modified food or food ingredient capable of providing health benefits beyond the basic nutrients it contains [7]. Notable examples include probiotic yoghurts enriched with specific bacterial strains that enhance gut health, Omega-3 fortified eggs that support cardiovascular function and plant sterol-enriched margarine that help reduce cholesterol levels [8].

Pasta serves as an excellent vehicle for nutritional fortification due to its composition of high-quality durum wheat semolina (*Triticum durum*), which typically contains 12%–13% protein (primarily gluten), 70%–75% complex carbohydrates, 1%–2% lipids and various micronutrients including B vitamins and minerals such as iron and magnesium [1]. This nutritional foundation, combined with pasta's neutral flavour profile, structural stability during processing and widespread consumer acceptance, makes it particularly suitable for nutritional enrichment. The enrichment is justified by the opportunity to address specific nutritional gaps in modern diets, particularly regarding essential amino acids, Omega-3 fatty acids and bioactive compounds, whilst simultaneously reducing food waste through by-product valorisation. The term ‘enriched pasta' refers to composite pasta products that incorporate one or more additional ingredients such as gluten, soy, eggs, milk, vegetables, legumes and various extracts or juices. Furthermore, other foodstuffs may be incorporated, provided they are authorised by the Directorate General of Public Health, in accordance with the provisions of Decree 2181/1975 [9]. Researchers have explored the enrichment of pasta with various food by-products, essential oils, alternative cereals and animal protein sources to enhance its nutritional profile and gastronomic diversity [7].

Pasta production involves both mechanical processes (mixing and extrusion) and thermal treatment (drying) that can potentially reduce the bioactive compound levels in the final product. Research has shown that these processes can lead to the degradation of sensitive compounds, such as polyphenols and antioxidants [6]. Functional ingredients must be carefully calibrated to maintain the stability and shelf life of the product, as exposure to high temperatures can diminish the bioactivity of certain nutrients. Modern consumers not only seek health-promoting qualities but also sensory experiences that evoke traditional flavours and textures [7]. In this context, enriched pasta not only meets the expectation of additional health benefits but also provides the organoleptic quality of the traditional product.

Innovative pursuit of food solutions aligns with broader industry goals of reducing food waste and advancing circular economic principles. The food industry faces increasing pressure to reduce waste and adopt sustainable practices. In this context, fish consumption has been growing steadily in recent years, with a significant global increase predicted for the next decade, with per capita consumption estimated at 21.2 kg by 2032 [10]. There is a clear need for fish by-product valorisation, as fish processing generates a considerable amount of waste, ranging from 30% to 50% of the original product [11].

Fish and fish by-products offer significant nutritional benefits, primarily due to their content of polyunsaturated fatty acids (PUFAs), particularly Omega-3 fatty acids, including *α*-linolenic acid (ALA), eicosapentaenoic acid (EPA) and docosahexaenoic acid (DHA). Additionally, they provide high-quality protein, essential amino acids, bioactive peptides, vitamins (A, D, B6 and B12) and minerals (phosphorus, magnesium, iron, zinc, selenium and iodine). Despite these benefits, fish consumption faces resistance amongst younger populations primarily due to negative taste preferences, a perceived lack of convenience and limited knowledge about the benefits of consuming fish [4, 12, 13]. Studies have shown that younger people often associate fish with strong, undesirable flavours or unpleasant textures, leading to reduced incorporation into their diet [4]. Moreover, the fish perception as difficult or time-consuming to prepare further contributes to its low popularity in this demographic [13]. These factors highlight the need for innovative approaches to make fish more appealing to younger consumers, particularly through functional development and easy-to-consume fish-based products [12].

One promising approach to using fish by-products involves converting them into functional ingredients such as fish meal or fish hydrolysates (FHs). Hydrolysis is an enzymatic process in which the by-products (skin, bones, head, spines or guts) are broken down into peptides, followed by heat treatment to inactivate the enzymes and standardise the fat content. This application of by-products is presented as a viable strategy for enriching existing foods with essential nutrients [11, 14]. It is fundamental to ensure that these new ingredients not only improve the nutritional value of final products but also maintain their quality, palatability and consumer appeal, particularly in the case of foods such as pasta. Palatability refers to the sensory attributes of a food, including taste, texture, aroma and appearance, which contribute to the overall eating experience. Pasta, texture factors, smoothness after cooking and flavour are essential for consumer acceptance. Whilst functional ingredients like fish protein hydrolysates or bioactive compounds can enhance the nutritional profile, these must not compromise the sensory qualities of the final product. Balancing nutritional benefits with palatability is key to ensuring the acceptance of enriched products in the marketplace [7, 15]. Diets rich in Omega-3 fatty acids have demonstrated potential in reducing chronic diseases such as cardiovascular disease, diabetes, cancer and obesity [4], underscoring the importance of efficient and sustainable use of fish by-products in food applications. The present study is aimed at evaluating the impact of incorporating FH at varying concentrations on the technological properties and nutritional value. The study variables include technological properties, nutritional parameters and stability characteristics. By investigating these variables, this study seeks to contribute to nutritionally developed enhanced pasta products that harness the health benefits of fish by-products whilst meeting consumer expectations for quality and palatability.

## 2. Materials and Methods

**Figure 1:**
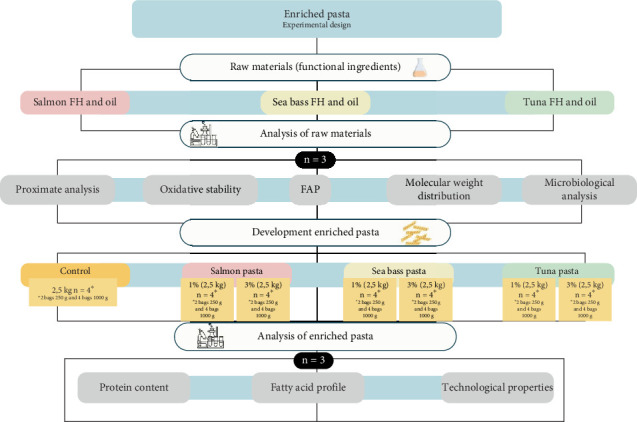
Experimental design of new enriched pasta with fish (salmon, sea bass and tuna). FAP, fatty acid profile.

An experimental design was carried out (Figure 1) for the enriched pasta. A two-factor factorial design (3 × 2) was used, with the two factors being the source of FH, three fish species and the concentration of FH (1% and 3%). The control samples consisted of pasta without the addition of FH. To meet the required nutritional fat content for the nutritional claim, 0.8% of FO from each FH source was added to enrich the pasta with PUFAs. The amount of FO added was calculated based on the Omega-3 content of the fish species used, ensuring that the final fat composition met the criteria for a nutritional claim. Each treatment was analysed in triplicate (*n* = 3) for proximate analysis, oxidative stability, molecular weight (Mw) distribution, fatty acid profile (FAP) and microbiological analysis. Additionally, pasta samples were evaluated for protein content, FAP and technological properties. All pasta treatments were packed in four bags (two of 1 kg and t of 250 g) and stored at −18°C until further analysis.

### 2.1. Materials

Durum wheat semolina (*Triticum durum*) was used to produce the enriched pasta. This semolina was supplied by Pastas Romero (Zaragoza, Spain). FHs from fish by-products were used as ‘flavouring preparation' according to Regulation 1334/2008 [16]. Specifically, hydrolysates from three fish species—salmon (*Salmo salar*), tuna (*Thunnus alalunga*) and sea bass (*Dicentrarchus labrax*)—were provided by the University of Zaragoza and were prepared following the method outlined by Honrado et al. [11].

Honrado et al. [11] produced FHs through an enzymatic hydrolysis process using Protamex and Alcalase enzymes. Fish heads were selected as the raw material, and enzymatic hydrolysis conditions—specifically temperature and pH set to the enzymes' optimal values, and time extended until the end of hydrolysis (indicated by a stable pH)—were optimised to ensure an acceptable sensory profile and a high yield of bioactive peptides. The process also involved a step of centrifugation to separate the oils from the hydrolysates. After hydrolysis, the FH and FO were stored at −18°C until their use.

### 2.2. Preparation of Enrichment Pasta

Six pasta prototypes were developed alongside a control formulation made with durum wheat semolina (*Triticum durum*) and water. The prototypes varied in the proportions of FH, derived from three fish species, at concentrations of 1% and 3%. Additionally, FO obtained from the FH was incorporated to enhance the PUFA content. The optimal water amount was determined during production, as FH is a dense liquid due to its protein-rich concentrate composition and protein content, which requires proper dilution to ensure homogeneous distribution within the dough. The quantities of FH and FO used are detailed in Table 1. The pasta was extruded in a fusilli format using a Bottene Mod. Lilodue CE extruder (Marano, Italy), which was chosen for its popularity in salads. The process commenced with mixing the dry ingredients (semolina), followed by the gradual addition of FH and water. The final product was packaged in sealed bags and stored at −18°C to maintain its microbiological stability and prevent degradation of its nutritional and sensory properties.

### 2.3. Characterisation of FH and FO

Physical analyses were conducted to evaluate the stability of the FH, specifically by determining its moisture content and water activity (*a*_w_). Moisture content was determined using a gravimetric method, following standard procedures. Briefly, approximately 5 g of each FH sample was accurately weighed in predried and tared aluminium dishes. The samples were then dehydrated in a forced-air drying oven at 105°C (Memmert, Germany) for at least 16 h, or until a constant weight was reached. After drying, the samples were cooled in a desiccator for 30 min and then reweighed. Moisture content was calculated as the percentage loss in weight relative to the initial mass.

*a*_w_ was measured using a hygrometric method with a Decagon Devices CX-1 (Washington, EE. UU). All measurements were carried out at room temperature and repeated in triplicate.

#### 2.3.1. Proximate Analysis of the FH

A proximate analysis of the FH was conducted to evaluate its proximate composition, specifically focusing on protein, fat and ash content, as described by Wani and Uppaluri [17].

The protein content was determined using the Kjeldahl method, according to AOAC [18] guidelines, as described by Honrado et al. [11]. Briefly, approximately 1 g of homogenised FH was digested with concentrated sulphuric acid and a catalyst tablet at 420°C. After cooling, the digest was subjected to distillation with sodium hydroxide (30%) (Carlo Ebra, Barcelona, España), and the released ammonia was trapped in a boric acid solution and titrated with standardised hydrochloric acid (Carlo Ebra, Barcelona, España). The nitrogen content was converted to protein using a specific nitrogen-to-protein conversion factor of 6.25, as recommended for FHs.

The total fat content was analysed by solvent extraction. The determination was carried out with a semiautomatic Soxhlet extractor (Selecta , DET-GRAS N, España) according to the AOAC method [19]. Approximately 3–5 g of freeze-dried FH was wrapped in a filter paper and placed into the extractor, where fat was extracted using petroleum ether as the solvent. The extracted fat was recovered by evaporation of the solvent, and the residue was weighed. This method aligns with the procedure applied by Ainsa et al. [3, 20] for similar fish-based products.

The ash content was determined gravimetrically following AOAC method 923.03 (2005). Samples were first dehydrated and ground to particles smaller than 1 cm in diameter to ensure uniform combustion. Approximately 2 g of sample was weighed into preweighed porcelain crucibles and incinerated in a muffle furnace at 500°C for 12 h, or until a white, constant-weight residue was obtained. This procedure allowed quantification of the total mineral residue present in each FH.

#### 2.3.2. Microbiological Analysis of Ingredients (FH)

These analyses were conducted to detect the presence of microorganisms that are important for both technological processing and hygienic safety, following protocols from UNE-EN ISO standards. Samples were prepared by suspending 10 g in 90 mL of peptone water (Merk, Darmstadt, Alemania) and homogenising the mixture to UNE-EN ISO 6887-2:2017 [21]. Three serial dilutions were prepared at 10^−1^, 10^−2^ and 10^−3^ (corresponding to 1:10, 1:100 and 1:1000). Microbial counts were performed using the 10^−2^ dilution. Readings were taken after incubation at appropriate temperatures, using necessary dilutions. Total viable count (TVC) at 30°C [22], mould and yeast counts (MY) (ISO 21527-1:2008) [23] and *Enterobacteriaceae* (ET) counts at 37°C (UNE-EN ISO 21528-2:2018) [24] were performed. Counts were recorded 72 h and 24 h later for TVC and ET and 3–5 days later for MY. Microbiological results were converted to log CFU/g after counting plates with 30–300 colonies.

#### 2.3.3. Oxidative Stability of FO

##### 2.3.3.1. Acidity Value

The acidity value was assessed following the guidelines outlined in ISO 660:2020 [25]. Subsequently, 10 mL of a neutralised ethanol solution was added, and the resulting supernatant was collected. The addition of phenolphthalein served as an indicator. Subsequently, the samples were titrated with a 0.1 N NaOH solution. Equation (1) was applied, where *V* represents the volume of NaOH solution used for titration (mL), *N* is the normality of the NaOH solution (eq/L), *m* is the mass of the sample (g) and 28.2 is the conversion factor used to express the result as % oleic acid.

(1) $$\begin{array}{c} \text{Acidity value }\left(\%\text{oleic acid}\right)=\frac{V\cdot N\cdot 28.2}{m}. \end{array}$$

##### 2.3.3.2. Peroxide Index (PI)

The PI was assessed following ISO 3960:2017 [26] (modified) guidelines. Initially, 0.5 g of the sample was weighed into a 100-mL Erlenmeyer flask. Subsequently, it was mixed with 7.5 mL of glacial acetic acid, 5 mL of n-hexane (Carlo Erba, Sabadell, Spain) and 0.2 mL of saturated KI (PanReac, Ottoweg, Germany). Stirring the solution for 2 min facilitated thorough mixing of the reagents. Following this, 25 mL of distilled water was added, and 1% starch (PanReac, Ottoweg, Germany) was employed as an indicator. The sample acquired a greyish colouration and was then titrated with 0.002 N sodium thiosulfate. Equation (2) used to calculate the PI was as follows:

(2) $$\begin{array}{c} \text{Peroxide index }\left(\text{meq }{\text{O}}_{2}/\text{kg}\right)=\frac{\left(V\cdot N\cdot 1000\right)}{m}, \end{array}$$

where *V* represents the volume of sodium thiosulfate (Na₂S₂O₃) consumed during titration (mL), *N* is the normality of the sodium thiosulfate solution (eq/L), *m* is the mass of the sample (g) and 1000 is the conversion factor to express the result as meq O₂/kg.

##### 2.3.3.3. TBARS Index

Lipid oxidation (TBARS) was assessed following the method outlined by [27]. The procedure involved constructing a calibration curve using 1,1,3,3-tetramethoxypropane (TMP) to quantify the malondialdehyde (MDA) content. Initially, 2 g of the sample was extracted and homogenised with concentrated trichloroacetic acid (TCA). After centrifuging samples at 4°C for 30 min at 4000 rpm, the thiobarbituric acid (TBA) reaction was performed in a thermostatic bath at 97°C for 20 min. Following sample cooling, absorbance was measured at 532 nm using a spectrophotometer (ONDA UNICAM, 5625 UV/VIS, China). This absorbance is compared to a calibration curve made with known MDA standards. The MDA concentration is extrapolated from the curve using the equation of the line. TBARS values are commonly expressed as micromoles of malondialdehyde per gram of sample or milligrams of malondialdehyde per kilogram of sample. In high-fat samples, results may be normalised per gram of fat. It is important to specify the basis (fat or total sample) for accurate interpretation.

### 2.4. FAP (Percentage)

The FAP was determined following the Bligh and Dyer [28] method, with modifications made to the analysis protocol in accordance with the procedure outlined by Ainsa et al. [3]. This modification was deemed necessary to suit different FOs better. Each sample (10 g) was homogenised in a mixture of 20 mL chloroform, 20 mL methanol and 14 mL water (*v*/*v*) using an Ultraturrax device (IKA-WERKE, T-25 basic, Germany) at 4000 rpm for 15 min at room temperature, following the protocol adapted from Ainsa et al. [3]. Solvents were evaporated under N_2_ current, with butylated hydroxytoluene (BHT) added as an antioxidant. Following this, methylation was carried out by adding 2 mL of n-hexane (Carlo Erba, Sabadell, Spain) and 1 mL of saturated potassium hydroxide. Fatty acids were quantified as the total area (%) of identified fatty acids (UNE-EN ISO 12966-2:2017 [29] modified).

### 2.5. Mw Distribution FH

The molecular mass of FH proteins was determined using size exclusion chromatography coupled with high-performance liquid chromatography (SEC-HPLC), a robust analytical technique for precise protein Mw estimation. This method leverages the principle of separating proteins based on their hydrodynamic size, which provides a highly reliable correlation with molecular mass. The experimental protocol was developed following the methodology proposed by Hong et al. [30], ensuring a standardised and reproducible approach to protein characterisation. Mw calibration was performed using protein standards ranging from 1.35 to 669 kDa (e.g., thyroglobulin, *γ*-globulin, ovalbumin, myoglobin and vitamin B12; Sigma-Aldrich), enabling accurate estimation of the molecular size distribution of the protein hydrolysates.

### 2.6. Technological Properties of Enriched Pasta

#### 2.6.1. OCT

The OCT of enriched pasta was assessed through a Warner–Bratzler cut test, following the guidelines provided in the instruction manual of the texturometer employed (Stable Micro Systems, ANAME, TA-CT2i, England). The OCT determination was conducted using a flat Warner–Bratzler attachment. Hardness determination involved assessing various sample durations, where hardness was defined as the maximum force (at a tangential angle) required to cut the sample, expressed in kilograms per second. The test conditions included the following: pretest speed: 2 mm/s; test speed: 2 mm/s; posttest speed: 10 mm/s; cutting distance: 15 mm; and threshold strength: 0.010 kg [3]. The load cell used a 5-kg capacity load cell, and the sample dimensions were 15 × 3 mm for optimal analysis.

#### 2.6.2. Moisture (Percentage)

Moisture content was determined using a gravimetric technique. The pasta samples were initially weighed and subsequently dried in a stove set at 105°C until reaching a consistent weight. After cooling to room temperature, the samples were weighed again. The moisture content was calculated using the following formula:

(3) $$\begin{array}{c} \text{Moisture}\left(\%\right)=\frac{\text{raw pasta weight}-\text{dried pasta weight}}{\text{raw pasta weight}}\times 100. \end{array}$$

#### 2.6.3. Weight Gain and Hydration

The increase in weight was determined by cooking 3 g of pasta in 180 mL of water in the OCT. Then, cooked pasta was allowed to cool at room temperature between moistened paper towels and weighed on an analytical balance. This parameter was calculated using the following formula:

(4) $$\begin{array}{c} \text{WG}\left(\%\right)=\frac{\text{Weight of cooked pasta }\left(\text{g}\right)-\text{Weight of raw pasta}\left(\text{g}\right)}{\text{Weight of raw pasta }\left(\text{g}\right)}\times 100. \end{array}$$

The cooked pasta underwent dehydration in an oven at 105°C for 24 h. The swelling index (SI) was then calculated using a specific mathematical equation:

(5) $$\begin{array}{c} \text{SI}=\frac{\text{Weight of cooked pasta }\left(\text{g}\right)}{\text{Weight of dried cooked pasta }\left(\text{g}\right)}\times 100. \end{array}$$

#### 2.6.4. Cooking Losses

A portion of 3 g of each pasta sample was taken to boiling with 180 mL of water and cooked for the optimal duration. The water remaining after cooking was gathered in crucibles and evaporated on a stove set at 105°C until a constant weight was achieved. The residue was then weighed and expressed as a percentage of the total weight of the raw pasta.

#### 2.6.5. Pasta Colour

The colour assessment of cooked pasta in OCT was conducted using a colourimeter (Minolta, CM-2002, Japan). The CIE Lab⁣^∗^ system, which includes *L*^∗^ (brightness), *a*^∗^ (redness) and *b*^∗^ (yellowness), was employed. The colour variation induced by each fish species was determined by calculating the total colour difference (Δ*E*) between the control pasta and the salmon, sea bass and tuna pasta:

(6) $$\begin{array}{c} \Delta E=\sqrt{{\left(\Delta L^{*}\right)}^{2}+{\left(\Delta a^{*}\right)}^{2}+{\left(\Delta b^{*}\right)}^{2}}, \end{array}$$

where Δ*L*^∗^ = *L*^∗^ fish pasta − *L*^∗^ control pasta; Δ*a*^∗^ = *a*^∗^ fish pasta − *a*^∗^ control pasta; and Δ*b*^∗^ = *b*^∗^ fish pasta − b^∗^ control pasta.

### 2.7. Protein Content of Enriched Pasta

A protein content analysis of the enriched pasta was conducted to verify the increase in protein content. The analysis was performed as described in Section 2.3.1.

### 2.8. FAP of Enriched Pasta Assays

A FAP analysis of the pasta was carried out using the method described in Section 2.4.

The atherogenicity index (AI) and thrombogenicity index (TI) were calculated by applying the mathematical formula developed by Ulbright and Southgate [31]:

(7) $$\begin{array}{c} A\text{I}=\frac{\left(\text{C}18:0\right)+\left(4\times \text{C}14:0\right)+\left(16:0\right)}{\left(\text{PUFA n}‐6\text{ and n}‐3\right)+\text{MUFA}}, \end{array}$$

(8) $$\begin{array}{c} \text{TI}=\frac{\left(\text{C}14:0\right)+\left(\text{C}16:0\right)+\left(\text{C}18:0\right)}{\left(0.5\times \text{MUFA}\right)+\left(0.5\times \text{PUFA n}‐6\right)+\left(3\times \text{PUFA n}‐3\right)+\left(\text{PUFA n}‐3/\text{n}‐6\right)}, \end{array}$$

where MUFA is the monounsaturated fatty acid and PUFA is the polyunsaturated fatty acid.

### 2.9. Statistical Analyses

The results of this study were analysed using XLSTAT Version 2016 (Addinsoft, Paris, France) [32]. For the FH and FO characterisation results, a univariate analysis was performed to verify the normality of the data and to detect outliers. Subsequently, a statistical analysis was applied by multifactorial ANOVA considering the different FH. To identify significant differences between the means of physicochemical, oxidative stability and Mws, Fisher's *a posteriori* test with a 95% confidence interval was used. As for the fatty acid content in the different enriched pasta and FH, a statistical analysis by ANOVA was also used, followed by Fisher's test with the same confidence interval to evaluate differences between means. Finally, the technological properties of the pasta were analysed with the same statistical approach, using multifactorial ANOVA and Fisher's *a posteriori* test, to identify variations in physical and chemical measurements, oxidative stability and Mws.

## 3. Results and Discussion

### 3.1. Characterisation of FH and FO

FH from sea bass, salmon and tuna showed significant differences (*p* ≤ 0.05) in their nutritional composition (protein, fat and ash) and physicochemical parameters (*a*_w_ and moisture), indicating the influence of each kind of species over the components and properties of the developed products (Table 2). Thus, the nutritional composition of the fish by-products affected the final quality of the FH, where factors such as enzyme type, pH, incubation time and process temperature played an important role, as a previous study has shown [33]. These findings align with observations by Chalamaiah et al. [34], who demonstrated that species-specific differences significantly impact the hydrolysate characteristics.

The study highlights significant variability in the composition of FH derived from sea bass, salmon and tuna. The protein content ranges from 35.32% in tuna to 39.31% in salmon, which is notably lower than the values reported in the literature. This discrepancy could be attributed to differences in hydrolysis conditions and enzyme specificity, as suggested by Kristinsson and Rasco [35], who demonstrated that proteolytic enzyme selection significantly influences the degree of hydrolysis and resultant protein content. The fat content was also low, ranging from 1.67% to 4.66%, whilst the ash content was between 4.17% and 6.75%. These findings are consistent with observations by Halim et al. [36], who noted that the centrifugation step typically employed during hydrolysate production can significantly reduce lipid content.

A critical finding of the study is the extremely high *a*_w_ values, which range from 0.880 to 0.929. These values far exceed the recommended stability limits of 0.50–0.60 established by Labuza and Altunakar, [37] for protein hydrolysates. Moisture content is closely related to these high *a*_w_ values, ranging from 51.96% to 57.21%. This high moisture content may compromise the product's shelf life and increase the risk of microbiological contamination.

These compositional differences highlight the complex interactions between raw materials characteristics, enzymatic processes and processing conditions, supporting the observations of Benjakul et al. [38] regarding the multifactorial nature of FH production. In comparison to existing literature, the FH study shows notable compositional variations. The protein content (35.32%–39.31%) is significantly lower than previously reported values (82.8%–91.90% by [39, 40]). The lipid content (1.67%–4.66%) aligns with findings from Opheim et al. [41] and Sripokar et al. [40], suggesting similar lipid extraction efficiencies during processing. The ash content (4.17%–6.75%) is consistent with prior studies, but the *a*_w_ values (0.880–0.929) differ significantly from established ranges (0.192–0.34 by [42] and [43]). This disparity could be attributed to insufficient dehydration during processing, a critical factor highlighted by He et al. [44] in their study on stability parameters of protein hydrolysates.

### 3.2. Microbiological Analysis of FH

The microbiological analysis of the FH demonstrated the absence of Enterobacteria, moulds and yeasts in all samples. However, considerable levels of the TVC were detected, with values of 4.71 log CFU/g in salmon, 4.50 log CFU/g in tuna and 3.99 log CFU/g in sea bass. The TVC count, particularly in the salmon FH, could be a consequence of the handling of the raw material before the hydrolysis process, particularly in species commonly utilised in food preparations such as sushi that necessitate multiple handling steps. This observation aligns with the findings by Møretrø et al. [45], who documented significant variations in microbial contamination during seafood processing. These findings were slightly higher than those determined by Gómez and Zapata [46] in red tilapia FH, where a notable reduction in microbial load was observed during the hydrolysis process, reporting initial microbial counts of TVC (3.71 log CFU/g), total coliforms (2.65 log CFU/g) and moulds and yeasts (2.30 log CFU/g) which were eventually reduced in the final hydrolysate (TVC: 2.56 log CFU/g; total coliforms: 1.65 log CFU/g and moulds and yeasts: 1 log CFU/g).

This reduction was attributed to the hydrolysis process conditions (45°C, pH 7), which were found to act as effective microbiological barriers. Similar antimicrobial effects of alkaline hydrolysis were documented by He et al. [44], who demonstrated that extreme pH conditions contribute significantly to microbial reduction in protein hydrolysates. According to the above, Roldán et al. [47], in previous research with anchoveta (*Engraulis ringens*) FH, indicated that the effective management of production variables and the freshness of the raw material were relevant factors in the reach of safe products. This is consistent with observations by Galla et al. [48], who emphasised the importance of implementing hazard analysis critical control point (HACCP) systems specifically designed for hydrolysate production. Furthermore, Kristinsson and Rasco [35] highlighted that standardisation of hydrolysis parameters not only optimises functional properties but also contributes significantly to the microbiological safety of the final products.

### 3.3. Oxidative Stability of FO

Lipid oxidation indicators (Table 3) showed significant differences (*p* ≤ 0.05) amongst the three types of hydrolysates studied. The PI, an indicator of primary oxidation, was highest in tuna FH (7.52 meqO_2_/kg), followed by sea bass (4.80 meqO_2_/kg) and salmon (2.43 meqO_2_/kg). On the other hand, the acid value (AV), which reflects secondary oxidation, showed a different pattern: the FH from sea bass had the highest value (2.74% oleic acid), whereas salmon and tuna had significantly lower values (0.53% and 0.63% oleic acid, respectively). These results suggested different levels of lipid degradation in each species. Tuna FH with high PI but low AV indicated a predominant primary oxidation phase characterised by the formation of hydroperoxides. In contrast, sea bass FH, with a moderate PI but the highest AV, indicated a lower presence of free fatty acids, secondary degradation products. Salmon FH, with the lowest values for both indices, showed the best oxidative stability amongst the three species studied, which corroborates findings by Eymard et al. [49] regarding the superior oxidative stability of salmon compared to other marine species.

The TBARS analysis revealed significant differences in the degree of lipid oxidation between the species under study, with tuna FH exhibiting the highest value (7.41 mg MDA/kg), followed by sea bass (2.78 mg MDA/kg) and salmon (1.08 mg MDA/kg). These findings are particularly remarkable given that values exceeding 2 mg MDA/kg have been linked to sensory rejection due to perceptions of rancidity in enriched pasta with fish [3]. This threshold is consistent with observations by Chaijan et al. [50], who established similar limits for consumer acceptability in fish-based products. These high values detected could be due to the following factors: the elevated polyunsaturated FAP characteristic of blue fish (tuna), exposure to pro-oxidant conditions during processing (temperature, oxygen, metal ions, etc.) and variations in natural antioxidant content between species.

Regarding the influence of processing conditions, Morales-Medina et al. [51] demonstrated that metal ions released during hydrolysis can catalyse lipid oxidation reactions, which may explain the elevated TBARS values observed in tuna hydrolysates. These findings suggest a direct implication for the quality of the final product, as the development of secondary oxidative compounds can negatively affect organoleptic characteristics and nutritional value, as well as potentially compromising food safety, a concern previously highlighted by Baron et al. [52].

To improve oxidative stability, especially in the case of tuna, strategies such as the incorporation of natural antioxidants, such as rosemary extracts [20], the optimisation of processing conditions (temperature control and minimisation of oxygen contact) and the use of packaging systems with barrier properties against oxygen and light are recommended. This approach is supported by research from Secci and Parisi [53], who demonstrated that multilayered active packaging can significantly extend the oxidative stability of fish-derived products.

### 3.4. Mw Distribution FH

**Figure 2:**
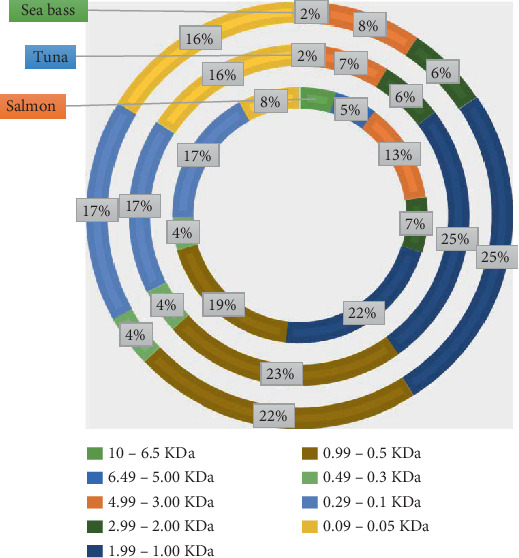
Molecular weight distribution (kDa) in FH. External circle: sea bass; medium circle: tuna; and internal circle: salmon.

A Mw analysis distribution revealed distinct patterns amongst the FH of the three species studied (Figure 2).

Distinct Mw profiles for the hydrolysates from different kinds of fish suggested that parameters such as distinct enzymatic activity, substrate specificity and protein composition could have a significant influence on the Mw distribution, as in a previous study [54]. The higher proportion of peptides in the 10–6.5 kDa range observed in salmon FH (4.15%–11.55%) compared to sea bass and tuna indicates less extensive hydrolysis, as can be seen in Figure 2. A larger peptide in this range is likely a remnant of the parent protein structure, suggesting that the enzymes used had limited activity on these protein sequences. This could be attributed to the presence of tightly folded domains in salmon proteins or differences in substrate accessibility [55].

Considering the above, Kim and Wijesekara [56] suggested that high-molecular-weight peptides (HMWPs) were often associated with gelling properties and could enhance the texture and stability of food matrixes. Therefore, the structural characteristics of salmon FH make it particularly suitable for applications requiring thickening or texturising agents, such as in meat substitutes or high-protein pasta. About intermediate-molecular-weight peptides (IMWPs) (3–4.99 kDa), the dominance of this fraction in salmon suggests a moderate degree of hydrolysis, which aligns with its application in products requiring enhanced water retention and emulsifying capacity [57].

These medium-sized peptides might contribute to the mouthfeel and overall organoleptic properties in food by increasing or adding a balanced umami or savoury flavour, particularly when used as a protein supplement [58]. Concerning low-molecular-weight peptides (LMWPs) (0.99–0.05 kDa), a higher proportion was found in tuna and sea bass FH (15.55%–25.05%). This suggests more extensive proteolytic breakdown. This high degree of hydrolysis could be due to the use of more forceful or specific enzyme preparations, which effectively cleave peptide bonds, producing smaller fragments [36]. Consequently, smaller peptides (< 1 kDa) are generally associated with enhanced bioactivity, including antioxidant and antihypertensive properties, due to their ability to interact with biological targets, such as enzymes or free radicals [59]. These bioactive peptides can be particularly beneficial when used in functional foods aimed at promoting cardiovascular health.

Based on our findings, distinct Mw distributions established across the three fish species assayed have direct implications for their potential use. Salmon FH with larger peptides offers advantages in gel formation and structural stability for protein bars and high-protein snacks. Bioactive sequences like Gly-Ala-Glu-Arg-Pro support cardiovascular health formulations [13], though fewer small peptides may limit immediate bioavailability. Tuna hydrolysates featuring LMWPs indicate high proteolysis, improving bioavailability and absorption rates [56]. These characteristics make tuna hydrolysates ideal for nutraceutical applications and enhance antioxidant capacity, potentially extending food shelf life by reducing oxidative stress [60]. Sea bass hydrolysates present a balanced peptide distribution, offering versatile functional properties. Intermediate-sized peptides improve emulsifying and water-binding characteristics, supporting functional food applications that require both texture and bioactivity [61]. Peptide size significantly influences sensory attributes: Smaller peptides (< 1.5 kDa) contribute umami flavours, whilst larger peptides (2–6 kDa) often introduce bitter notes [57]. Therefore, the higher proportion of low Mw peptides in tuna and sea bass hydrolysates could enhance the taste profile of food products, making them more appealing as flavour enhancers. In addition, the presence of peptides in specific Mw ranges, such as those high in lysine and threonine, could address common amino acid deficiencies in plant-based diets, thus enriching the nutritional profile of products like fortified pasta [62].

Analysis of the Mw distribution in FH from salmon, tuna and sea bass highlights the importance of selecting specific FH based on the desired functional and nutritional properties. The salmon FH was suitable for textural applications, whilst the tuna FH stood out for its bioavailability and antioxidant activity. Finally, the sea bass FH offered a versatile and balanced profile. Tailoring the choice of hydrolysate to the intended food application can optimise both product quality and consumer health benefits.

### 3.5. Technological Properties of Enrichment Pasta

#### 3.5.1. OCT

OCT revealed that pasta firmness progressively decreases with cooking time. The enriched pasta with FH demonstrated significantly reduced cooking times compared to conventional pasta. Samples with salmon and sea bass FH required only 150 s to reach optimal cooking, whilst those with 1% and 3% tuna extended slightly to 180 s. This substantial reduction from the typical 10-min cooking time for traditional pasta can be attributed to multiple factors: (1) the reduced proportion of starch available for gelatinisation, (2) the partial substitution of wheat semolina with highly soluble protein hydrolysates and (3) enhanced water absorption dynamics within the modified pasta matrix [3].

These findings confirm research by Khodaei et al. [63] found that fortifying foods with 10%–15% fish protein hydrolysate reduced cooking time from 10 to 8.53 min. They attributed this reduction to the disruption of the gluten network and the presence of low Mw components. Wójtowicz and Mościcki [64] corroborated this explanation, suggesting that protein additives can alter pasta's microstructure, facilitating more rapid water absorption and consequently shorter cooking times. Similarly, Desai et al. [4] established a direct connection between a weakened gluten network and decreased cooking time, whilst Ainsa et al. [65] associated this reduction with an increased presence of lower Mw compounds that facilitate faster hydration.

However, the relationship between protein enrichment and cooking time is not straightforward. Yang et al. [66] demonstrated that higher protein content may impede water penetration into starch granules by forming amylose–lipid complexes, potentially extending cooking duration. This effect was confirmed by Ramya et al. [67] and Liu et al. [68], who observed that increasing shrimp protein concentrations resulted in prolonged cooking times. Additionally, Ainsa et al. [3, 20] observed similar effects with marine proteins, suggesting that the source and composition of supplementary proteins significantly influence cooking behaviour. Enhanced water solubility accelerates absorption versus protein–starch interactions, restricting it.

#### 3.5.2. Weight Gain, SI and Cooking Losses

The analysis of the technological properties demonstrated that the FH incorporation significantly influenced the water-holding capacity of the dough (Table 4). The SI was notably reduced in fortified formulations, suggesting that protein–starch–lipid matrix interactions constrain water absorption during cooking. Although no statistical differences in WG were observed amongst the samples, pasta with 3% sea bass and tuna exhibited the highest values (172.66% and 166.38%), whilst the control sample had the lowest value at 123.74%. These differences suggest that water absorption capacity depends on the compositional factors, as interactions between lipids, proteins and starch can reduce hydration.

For the SI, all enriched pasta demonstrated a consistent reduction (ranging from 3.37% to 3.96%) compared to the control (8.78%), indicating lower water absorption. Fish protein incorporation appears to limit the pasta's SI during cooking. This finding contrasts with the SI value of 1.93% reported by Desai et al. [4] for a 5% enrichment, suggesting that the structural modifications induced by FH differ from those caused by intact proteins.

Regarding cooking losses, values ranged from 2.02% to 3.67%. This suggests that the inclusion of fish did not compromise the structural integrity of the pasta matrix. Pongpichaiudom and Songsermpong [69] proposed that certain marine proteins might reinforce the pasta structure through protein–protein interactions that compensate for gluten network disruption. This finding contrasts with the results reported by Desai et al. [4] led to increased losses due to more extensive alterations in the gluten network. This discrepancy may be explained by the difference in enrichment levels and the protein molecular characteristics of the sources used.

Moisture content revealed higher values (up to 22.68%) in pasta enriched with 1%–3% sea bass/tuna compared to the control (15.91%). This indicates that FH improves water-holding capacity during processing and storage, compared with the observations regarding WG and SI during cooking. Larrosa et al. [70] observed similar effects when incorporating protein hydrolysates in starch-based matrices, attributing this to the hydrophilic nature of peptides that can form hydrogen bonds with water molecules.

In summary, pasta water absorption capability was significantly influenced by its compositional profile. The interaction of fish lipids and proteins with starch during cooking affects hydration dynamics, resulting in complex changes to the cooking properties of the enriched pasta. These findings are linked with previous studies that have explored pasta enrichment with various fish and crustacean powders [3, 4, 67, 71]. The observed reduction in the SI of enriched pasta can be attributed to the reinforced protein network and complexes between starch and lipids, as suggested by Krishnan and Prabhasankar [72] in their comprehensive review of protein-enriched pasta products. Nevertheless, despite these modifications, the technological properties of enriched pasta formulations remain comparable to conventional pasta, demonstrating the feasibility of creating nutritionally enhanced pasta products without compromising fundamental cooking quality.

#### 3.5.3. Pasta Colour

**Figure 3:**
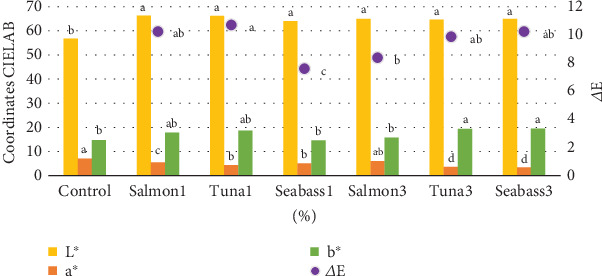
Colour parameters in the different types of pasta prototypes were developed. Different letters indicate significant differences (*p* < 0.05) between different parameters of treatments. *L*^∗^, brightness; *a*^∗^, redness; *b*^∗^, yellowness.

Pasta colour analysis revealed significant differences between the enriched pasta and control samples (Figure 3). Pasta made with FH exhibited higher brightness coordinates, likely due to the nature of the ingredients used, contrasting with the more yellowish hue of control pasta. FO inclusion also affected the yellow colour coordinates, an important factor in the sensory perception of the product. Although no statistically significant differences (*p* ≤ 0.05) in colour were found amongst the various enriched pasta, several notable trends emerged. Brightness levels remained relatively consistent across FH samples, whilst the control sample demonstrated lower brightness. This difference can be attributed to the lighter, almost white tones of the enriched samples compared to the yellowish tone of the control.

Regarding the red–green coordinates (*a*^∗^), the control showed a higher value, followed by the 3% salmon pasta. This may be due to the presence of salmon oil, which has orange tones from astaxanthin, a carotenoid extensively studied by Shahidi and Brown [73], for its chromatic properties in marine-derived products. The yellow–blue colour coordinate (*b*^∗^) was highest in enriched pasta with 3% tuna FH and 3% sea bass, followed by the samples with 1% sea bass and 1% salmon. Whilst less pronounced, this trend was also observed in other fortified pasta formulations. These results align with findings reported by Santeramo et al. [74], who found that incorporating marine proteins into cereal-based food matrices tends to modify chromatic parameters depending on the concentration and type of fish used.

The overall analysis of colour change (Δ*E*) revealed significant differences (*p* ≤ 0.05) between enriched pasta samples and the control. The pasta with 1% salmon had a Δ*E* of 10.24, whilst the 1% tuna sample had a slightly higher Δ*E* of 10.71. For the FH additions at 3%, the Δ*E* values were found to be 8.37 for salmon, 9.87 for tuna and 10.23 for sea bass. It is important to note that Δ*E* values greater than 2 are highly perceptible to consumers, as established by Mokrzycki and Tatol [75] in their studies on colour perception in food products. These findings suggest that FH incorporation may influence the colour of the pasta, although it is not the sole determining factor. The variations in Δ*E* values amongst the different samples can be attributed to the unique characteristics of the FH used and their interactions with the pasta's composition.

In the colour analysis of the enriched pasta, both brightness and the red index (*a*^∗^) showed similar trends, with the control pasta having the lowest brightness and the highest red colouring. This behaviour can be explained by the absence of colour-altering ingredients in the control pasta, resulting in a darker colour. Martínez-Sanz et al. [76] observed similar results when incorporating different types of marine-derived proteins into starch-based food systems, concluding that protein–carbohydrate interactions decisively influence the optical properties of the final product.

The yellow index (*b*^∗^) was a particularly prominent parameter, as consumers generally prefer bright yellow pasta. Khodaei et al. [63] suggest that this preference is linked to the association of yellow colour with higher product quality. In this context, pasta enriched with 3% tuna and 3% sea bass displayed a notably bright yellow colour, likely making it more appealing to consumers. Beyond yellow shades, there are also market preferences for other pasta colours, such as blue–green, which are especially noticeable in pasta that includes seaweed in its composition [65]. This chromatic diversification represents an opportunity to expand the range of functional products, as pointed out by Fradique et al. [77] in their study on the acceptance of pasta with different shades derived from marine ingredients. Additionally, Lucas et al. [78] have highlighted that colour changes in products enriched with marine proteins can be modulated through natural antioxidant use, which not only preserves desirable chromatic characteristics but also contributes to the oxidative stability of the product during storage.

### 3.6. Protein Content of Enrichment Pasta

The protein content in the different enriched dry pasta samples showed significant (*p* ≤ 0.05) changes depending on the type and amount of FH used. The control pasta had a protein content of 12.30%, which was the lowest, as expected because it is the usual content for pasta with durum wheat semolina. When 1% FH was added, the salmon-enriched pasta increased to a value of 13.24%. This was the highest at 1% in terms of protein content, significantly higher than those containing tuna FH (12.42%) and sea bass FH (12.58%), which did not differ significantly from each other. At a concentration of 3%, the pasta with salmon FH (15.45%) had the highest protein content, as did the pasta with tuna FH (15.33%) but significantly higher than the pasta enriched with sea bass FH (14.76%). When comparing concentrations, all pasta samples exhibited a protein content increase from 1% to 3%. Amongst the fortification methods, salmon FH proved to be the most effective at both concentrations, significantly enhancing the protein content.

Protein quality represents a key factor in determining pasta's nutritional characteristics and values. Spanish statute (Decree 2181/1975) [9] requires that the minimum protein content of plain dry pasta must be 9.5%. In this sense, the pasta produced in this study showed significantly increased protein content, exceeding both the minimum established by legislation and values reported in other studies. The variation in protein enhancement across different fish species can be attributed to the intrinsic differences in their muscle composition and protein profiles.

For instance, Khodaei et al. [63] obtained protein contents of 10.56, 14.74, 18.46 and 20.37 g/100 g of pasta in their dry pasta samples with 0%, 5%, 10% and 15% FPH, respectively. Likewise, Desai et al. [4] demonstrated that fish powder incorporation could increase the protein content, from 12.7% in the control to 29.8%in the sample with 20% fish protein. These increases are related not only to the direct addition of protein-rich material but also to the potential synergistic interactions between fish and cereal proteins that enhance overall protein retention, as investigated by Tazrart et al. [79] in their work on legume-enriched pasta formulations.

Similarly, Desai et al. [80] reported that salmon protein addition increased the protein content from 12.6% in the control samples to 22.7% in enriched pasta at 5%,10%,15% and 20%. Furthermore, in the study by Saini et al. [81] pasta enriched with protein isolate from *Pangasius hypophthalmus* demonstrated a progressive increase in protein content, rising from 14.52% to 20.79% as the protein addition was increased from 2.5% to 10%. Vijaykrishnaraj et al. [71] also observed similar trends when incorporating shellfish protein into pasta, noting that the functional properties of the pasta matrix were maintained despite substantial increases in protein content.

The effectiveness of protein enrichment observed in this study is particularly notable considering the relatively low incorporation rates (1% and 3%) compared to other studies that employed higher percentages. This efficiency may be attributed to the hydrolysed nature of the fish proteins used, which Chalamaiah et al. [34] have been shown to exhibit enhanced solubility and integration capabilities within food matrices compared to intact proteins. Moreover, Aspevik et al. [82] demonstrated that protein hydrolysates derived from fish by-products often contain a higher concentration of bioactive peptides and essential amino acids than those from prime cuts, potentially enhancing the nutritional value beyond what is reflected in total protein measurements.

### 3.7. FAP of FH and Enrichment Pasta

The analysis of the FAP revealed significant differences (*p* ≤ 0.05) in the fatty acid composition across studied treatments, with a particular focus on the impact of PUFAs, monounsaturated fatty acids (MUFAs) and saturated fatty acids (SFAs) (Table 5). The lipid profile analysis of the control and enriched pasta samples revealed significant differences (*p* ≤ 0.05) based on the FH species used. Notably, tuna and sea bass FH increased the content of SFAs, particularly myristic (C14:0) and palmitic acids (C16:0), with sea bass-enriched pasta showing the highest myristic acid levels (3.37%). These findings align with those reported by Turchini et al. [83], who documented species-specific FAPs in various fish species, highlighting the distinctive lipid composition patterns that can impact fortified food products.

In contrast, MUFAs were significantly elevated in pasta enriched with salmon and sea bass FH, driven mainly by oleic acid (C18:1 n-9). It is important to note that the origin of the species influences the final lipid profile: salmon and sea bass, as aquaculture species fed on a vegetable-rich diet, have higher levels of Ω6 fatty acids, mainly oleic and linoleic [3] which reached 30.01% in sea bass pasta compared to 13.18% in the control sample. Additionally, eicosenoic acid (C20:1) levels were notably higher in the enriched pasta, particularly in salmon FH (3.50%). Certainly, the fatty acid modification composition could modify the technological properties, as well as the nutritional characteristics of the enriched pasta, as previously documented by Gajanan et al. [84] in their comprehensive studies on marine lipid incorporation into cereal-based food matrices.

The most relevant contribution from a nutritional point of view was observed in PUFAs, especially Ω3. The pasta enriched with tuna was characterised by high concentrations of EPA (4.79%) and DHA (17.89%), significantly higher than the control. Amongst the samples, PUFA content varied markedly, with sea bass and salmon pasta samples showing the highest PUFA percentages (46.56% and 44.08%, respectively). PUFAs, including Ω3 and Ω6 fatty acids, are essential components of the diet due to their significant role in cardiovascular health, inflammation modulation and cognitive function, as extensively reviewed by Calder [85].

Unsaturated fatty acids of type Ω3, particularly EPA (C20:5 n-3) and DHA (C22:6 n-3), were substantially elevated in fish-enriched samples. For instance, tuna had the highest concentration of DHA (27.80%), which is a critical fatty acid for brain health and anti-inflammatory properties. Conversely, Ω6 fatty acids, such as linoleic acid (C18:2 n-6), were more prominent in the control and pasta samples, reflecting the influence of plant-based oils typically rich in omega-6 PUFAs.

Furthermore, the incorporation of FH significantly improved the Ω6/Ω3 ratio, which reached optimal values in the tuna (0.85) and sea bass (0.20) pasta compared to the control (6.22). These results are in line with those reported by Ainsa et al. [20] and Calanche et al. [86]. Regarding the above, the balance between Ω6 and Ω3 fatty acids is crucial for maintaining optimal health, as it influences the inflammatory response and overall cardiovascular risk [87]. This important balance was further emphasised by Patterson et al. [88], who conducted a meta-analysis demonstrating significant cardioprotective effects associated with a lower *Ω*6/*Ω*3 ratio in the diet.

In this study, the ratio (*Ω*6/*Ω*3) varied significantly amongst the samples, providing insights into their potential health impacts. A lower *Ω*6/*Ω*3 ratio is generally considered beneficial due to its association with reduced inflammatory responses [87]. The sea bass and tuna samples demonstrated the lowest *Ω*6/*Ω*3 ratios (0.09 and 0.11, respectively), suggesting a strong anti-inflammatory effect. These values contrast sharply with the control sample, which had a much higher *Ω*6/*Ω*3 ratio (6.22), indicating a greater potential for promoting inflammation, a risk factor for chronic diseases such as cardiovascular disease and metabolic syndrome. These results agree with those found in a similar study, confirming that enriching with unsaturated fats from salmon and tuna FH improves the *Ω*6/*Ω*3 ratio, which values to prevent chronic diseases are between 1:1 and 2:1 [11].

The PUFA/SFA ratio (P/S ratio) serves as an important measure of dietary fat quality, with higher values indicating a healthier FAP. In this analysis, the salmon and sea bass samples exhibited the highest P/S ratios of 2.35 and 2.10, respectively, reflecting their superior PUFA content relative to SFA. This ratio is a key indicator of lipid quality, as diets with higher P/S ratios are associated with improved heart health and a reduced risk of atherosclerosis [89]. In contrast, the control and sea bass pasta samples had lower P/S ratios of 1.80 and 0.60, respectively, suggesting a higher proportion of saturated fats, which can lead to negative health effects such as increased LDL cholesterol levels. Overall, this analysis highlights the significant nutritional benefits of enriched pasta, particularly in enhancing Omega-3 PUFA intake, improving the PUFA/SFA balance and reducing the Omega-6 to Omega-3 ratio. These factors are all favourable for promoting anti-inflammatory responses and cardiovascular health, as extensively documented in the systematic review by Abdelhamid et al. [90].

The significance of this is amplified by the well-documented benefits of EPA and DHA for cardiovascular and brain health. The findings of this study support this established connection and reaffirm the value of incorporating unsaturated fats from salmon and tuna FH. By adding these FH, a more balanced and nutritionally advantageous lipid profile is achieved. This approach optimises the Omega-6/Omega-3 ratio whilst increasing the essential levels of fatty acids like EPA and DHA, which are noted for their positive impacts on cardiovascular health and brain function, as comprehensively reviewed by Swanson et al. [91] in their analysis of the role of these fatty acids in human health and disease prevention.

Values for AI and TI ranged between 0.22 and 0.55, values consistent with those reported in the literature for fish species. To compare other foods like milk, which presents an index close to 1, these results are significantly lower. Diets with low AI and TI values are associated with a reduction in the potential risk of coronary diseases. Ulbricht and Southgate [31] in their pioneering work on these indices demonstrated this. More recently, Chen and Liu [92] confirmed the predictive value of these indices for cardiovascular disease risk assessment, particularly in the context of functional food development. The variability observed between species suggests that the specific lipid profile of each FH may influence these indices, which could have important implications for their potential use as a functional ingredient in foods aimed at preventing cardiovascular diseases. This observation is supported by the comprehensive analysis conducted by Strobel et al. [93], who documented species-specific variations in lipid composition and the resulting health implications.

## 4. Conclusion

Fish by-product incorporation in pasta production has proven to be an effective strategy for improving its nutritional profile significantly. This improvement is mainly due to three aspects: the increase in the content of high biological value proteins, the enrichment with Ω3 fatty acids (EPA and DHA) and the addition of bioactive peptides with health benefits. The optimisation of the Ω6/Ω3 ratio, together with the increase in the MUFA profile, represents a significant nutritional improvement over conventional pasta. From a technological point of view, the FH and FO addition has a positive impact on the product's functional properties. Better water retention and pasta reduction swelling were key factors that contributed to improving the quality and texture of the final product. Regarding the microbiological quality of raw material (FH), it was satisfactory but highlighted the need for rigorous controls in handling and by-product production.

A particularly relevant finding is the presence of bioactive peptides with potential antioxidants, antihypertensive and antimicrobial properties in FH, observed in the very low Mw fractions in the case of the antioxidant and antibacterial, and the low Mw fractions in the case of antimicrobials, which are of great interest to the food industry. These compounds may not only help to improve the oxidative stability of products but may also provide potential health benefits, thus supporting the revaluation of these by-products. In conclusion, this study demonstrates that FH is a viable and beneficial alternative for nutritionally developed food products. This application not only contributes to the development of healthier foods but also provides a sustainable solution to the problem of waste in the fishing industry, in line with the circular economy and sustainable food production principles.

Despite these promising results, several limitations and challenges were encountered during this research. Sensory acceptance remains a significant concern, as FH incorporation can contribute characteristic flavours that might affect consumer acceptance. Additionally, the stability of bioactive compounds during processing and storage requires further investigation to ensure their preservation throughout the product's shelf life. The variability in the composition of fish by-products depending on species, season and processing methods also presents challenges for standardisation. Furthermore, the cost-effectiveness of the extraction and hydrolysis processes needs to be optimised to make industrial-scale production economically viable.

Future perspectives for this research include further exploration of specific bioactive peptides identified in FH and their potential applications in functional foods beyond pasta products. Additional studies could focus on optimising extraction and hydrolysis methods to enhance the yield and bioactivity of these compounds. Consumer acceptance studies would also be valuable to determine market potential and guide product development strategies. Moreover, scaling up the production process whilst maintaining quality and safety standards represents an important challenge for industrial implementation. Collaboration between the seafood processing industry, food manufacturers and research institutions will be crucial to establish effective valorisation chains for fish by-products. Finally, regulatory frameworks for novel food ingredients derived from by-products will need to be addressed to facilitate commercial adoption of these sustainable and nutritionally enhanced products.

## Figures and Tables

**Table 1:** Formulations of enriched pasta with FH of different species: salmon, sea bass and tuna.

**Ingredients (%)**	**Pasta FH SA1%**	**Pasta FH SEA1%**	**Pasta FH TU1%**	**Pasta FH SA3%**	**Pasta FH SEA3%**	**Pasta FH TU3%**
Semolina	73.2	73.2	73.2	71.2	71.2	71.2
Salmon oil	0.8	—	—	0.8	—	—
Salmon FH	1	—	—	3	—	—
Sea bass oil	—	0.8	—	—	0.8	—
Sea bass FH	—	1	—	—	3	—
Tuna oil	—	—	0.8	—	—	0.8
Tuna FH	—	—	1	—	—	3
Water	25	25	25	25	25	25

Abbreviations: SA, salmon; SEA, sea bass; TU, tuna.

**Table 2:** Characterisation of salmon, sea bass and tuna FH (nutritional composition and physical parameters studied).

	**Protein (%)**	**Fat (%)**	**Ash (%)**	**a**_**w**_	**Moisture (%)**
Sea bass FH	36.00^b^	4.66^a^	4.95^b^	0.892^b^	51.96^c^
Salmon FH	39.31^a^	1.67^b^	4.17^b^	0.929^a^	54.65^b^
Tuna FH	35.32^c^	1.87^b^	6.75^a^	0.880^b^	57.21^a^
*Pr > F*	0.000	0.000	0.000	0.000	0.000

*Note:* Letters indicate significant differences (*p* < 0.05) amongst hydrolysates.

**Table 3:** Oxidative stability of FO of the studied species, salmon, sea bass and tuna. The lower-case letters indicate significant differences (*p* < 0.05) between the analysis of each FH and its corresponding FO.

	**PI (meqO**_**2**_ **/kg)**	**AV (% oleic acid)**	**TBARS (mg MDA/kg)**
Tuna oil	7.52^a^	0.63^b^	7.41^a^
Sea bass oil	4.80^b^	2.74^a^	2.78^b^
Salmon oil	2.43^c^	0.53^b^	1.08^c^
*Pr > F*	0.007	0.000	0.001

*Note:* Different letters indicate significant differences (*p* < 0.05) between FH and its corresponding FO.

**Table 4:** Values of technological properties for each analysed pasta.

	**WG (%)**	**SI (%)**	**CL (%)**	**M (%)**
Control	123.74	8.78^a^	2.02	15.91^c^
1% salmon	164.70	3.37^b^	3.34	18.25^bc^
1% sea bass	137.09	3.50^b^	3.23	22.00^a^
1% tuna	162.89	3.96^b^	2.69	22.20^a^
3% salmon	154.06	3.73^b^	2.15	22.68^a^
3% sea bass	172.66	3.64^b^	3.67	18.24^bc^
3% tuna	166.38	3.40^b^	2.42	20.25^ab^
*Pr > F*	0.802	0.000	0.726	0.004

*Note:* Distinct letters indicate significant differences (*p* < 0.05) amongst different pasta types for each technological property.

Abbreviations: CL, cooking losses; M, moisture; SI, swelling index; WG, weight gain.

**Table 5:** Fatty acid profiles for enriched pasta with FH.

	**Control**	**Salmon FH**	**Tuna FH**	**Sea bass FH**	**Salmon pasta**	**Tuna pasta**	**Sea bass pasta**
C12:0⁣^∗∗^	0.005^b^	0.005^b^	0.005^b^	0.005^b^	0.03^a^	0.03^a^	0.03^a^
C14:0⁣^∗∗∗^	0.09^g^	1.80^f^	2.50^c^	3.37^a^	2.66^b^	1.95^e^	2.13^d^
C16:0⁣^∗∗∗^	21.09^a^	8.70^f^	19.70^b^	14.20^e^	15.70^d^	19.20^b^	16.08^c^
C17:0⁣^∗∗∗^	0.13^f^	0.17^f^	1.30^b^	0.36^e^	1.04^c^	1.50^a^	0.49^d^
C18:0⁣^∗∗∗^	1.66^e^	2.30^f^	6.10^a^	2.58^e^	3.31^c^	4.26^b^	2.71^d^
C20:0⁣^∗∗∗^	0.15^c^	0.30^b^	0.48^a^	0.31^b^	0.19^c^	0.005^d^	0.005^d^
C21:0⁣^∗∗∗^	0.02^c^	0.005^c^	0.13^b^	0.86^a^	0.00^d^	0.005^d^	0.005^d^
C22:0⁣^∗∗∗^	0.03^c^	0.15^b^	0.39^a^	0.005^d^	0.005^d^	0.005^d^	0.005^d^
C24:0⁣^∗∗∗^	0.03^g^	0.06^f^	0.29^c^	0.47^b^	0.25^d^	0.58^a^	0.19^e^
% SFA⁣^∗∗∗^	23.30^c^	13.48^f^	30.89^a^	22.15^d^	23.15^c^	27.49^b^	21.60^e^
C16:1⁣^∗∗∗^	0.15^g^	2.00^e^	3.80^a^	1.50^f^	3.36^b^	2.71^d^	3.18^c^
C17:1⁣^∗∗∗^	0.06^g^	0.17^f^	0.86^a^	0.50^c^	0.47^d^	0.60^b^	0.25^e^
C18:1 n-9 (oleic)⁣^∗∗∗^	13.18^g^	42.00^a^	18.90^d^	15.34^f^	22.68^c^	17.89^e^	30.01^b^
C20:1⁣^∗∗∗^	0.02^g^	3.50^a^	1.30^f^	1.37^e^	3.03^c^	1.45^d^	3.34^b^
C22:1⁣^∗∗∗^	0.005^d^	2.63^a^	0.62^b^	0.25^c^	0.005^d^	0.005^d^	0.005^d^
C24:1⁣^∗∗∗^	0.15^d^	0.31^c^	1.00^a^	0.35^b^	0.005^e^	0.005^e^	0.005^e^
%MUFA	14.42^g^	50.61^a^	26.48^d^	19.31^f^	29.54^c^	22.65^e^	36.78^b^
C18:3 n-3 (ALA)⁣^∗∗∗^	4.08^b^	6.80^a^	0.51^e^	3.07^c^	2.29^d^	0.005^f^	2.97^c^
C18:2 n-6 (linoleic)⁣^∗∗∗^	35.87^a^	14.60^e^	16.40^d^	32.30^b^	17.08^d^	18.31^c^	0.16^f^
C20:2 n-6⁣^∗∗∗^	—	1.10^a^	0.31^d^	0.50^c^	0.31^d^	0.28^e^	0.60^b^
C20:3 n-6⁣^∗∗∗^	—	0.57^b^	0.12^e^	0.39^d^	0.07^f^	0.64^a^	0.50^c^
C20:4 n-6⁣^∗∗∗^	—	0.89^c^	2.10^a^	0.005^f^	0.85^d^	0.98^b^	0.51^e^
C22:6 n-3 (DHA)⁣^∗∗∗^	—	4.40^f^	27.80^a^	6.42^d^	13.81^c^	17.89^b^	4.58^e^
C20:5 n-3 (EPA)⁣^∗∗∗^	—	2.30^e^	5.40^a^	3.88^c^	1.40^f^	4.79^b^	2.49^d^
C22:5 n-3⁣^∗∗∗^	—	0.91^e^	2.00^a^	0.005^g^	1.40^b^	1.19^c^	1.06^d^
%PUFA⁣^∗∗∗^	42.03^bc^	31.67^e^	40.00^c^	46.56^a^	37.21^d^	44.08^b^	12.87^f^
Ʃ*Ω*3	5.8	14.41	35.71	10.30	18.90	23.87	8.13
Ʃ*Ω*6	36.12	17.16	3.93	0.89	18.31	20.21	1.61
P/S ratio	1.80	2.35	1.30	2.10	1.61	1.60	0.60
*Ω*6/*Ω*3 ratio	6.22	1.19	0.11	0.09	0.97	0.85	0.20
AI	0.46	0.55	0.54	0.47	0.22	0.44	0.46
TI	0.27	0.53	0.41	0.40	0.21	0.41	0.27

*Note:* Different letters indicate significant differences between the different parameters between treatments.

⁣^∗^*p* < 0.05, ⁣^∗∗^*p* < 0.01 and ⁣^∗∗∗^*p* < 0.001.

## Data Availability

The data that support the findings of this study are available from the corresponding author upon reasonable request.
